# Beyond Screening Populations: Context-Dependent Performance of Lung Cancer Risk Assessment Strategies in a Diagnostically Enriched Cohort

**DOI:** 10.3390/healthcare14193350

**Published:** 2026-10-08

**Authors:** Aysegul Erinc, Ali Kirac, Damla Azakli, Guliz Kizir, Gulsum Kirhan Karabulut, Hilal Rumeysa Ozkan Eksert, Cetin Yakisik, Celal Satici

**Affiliations:** 1 Department of Pulmonology, Yedikule Chest Diseases and Thoracic Surgery Training and Research Hospital, Health Sciences University, 34020 Istanbul, Turkey; 2 Department of Pulmonology, Basaksehir Cam and Sakura City Hospital, Health Sciences University, 34480 Istanbul, Turkey

**Keywords:** lung cancer, risk prediction, screening eligibility, PLCOm2012, Bach

## Abstract

**Background/Objectives:** Lung cancer screening with low-dose computed tomography has been shown to reduce disease-specific mortality, but optimal strategies for identifying high-risk individuals remain debated. This retrospective, single-center cohort study examined the context-dependent behavior of two individualized lung cancer risk prediction models, PLCOm2012 and the Bach model, and National Comprehensive Cancer Network (NCCN) screening criteria when retrospectively applied to hospitalized patients undergoing diagnostic evaluation. The study was not designed as an external validation of these approaches for their intended screening purpose, but rather to assess their ability to discriminate between patients with and without prevalent lung cancer in a diagnostically enriched clinical population. **Methods:** Among 254 patients in the overall cohort, 86 had histopathologically confirmed primary lung cancer. For the primary head-to-head comparison, a common analytic cohort of 133 current or former smokers for whom all three approaches could be evaluated was used; 56 of these patients had lung cancer. **Results:** PLCOm2012 demonstrated high sensitivity (87.5%) but low specificity (15.6%), whereas the Bach model, at the cohort-derived exploratory threshold of ≥0.49%, showed lower sensitivity (42.9%) and higher specificity (57.1%). NCCN criteria yielded the highest sensitivity (96.4%) but very low specificity (3.9%). Likelihood ratios were close to unity for all three approaches, indicating limited discrimination in this diagnostically enriched population. Paired analyses demonstrated significant differences in sensitivity and specificity between several of the threshold-based approaches, whereas continuous discrimination did not differ significantly between PLCOm2012 and the Bach model; overall discrimination remained limited in this diagnostically enriched cohort. **Conclusions:** These findings highlight the context-dependent performance of screening-oriented risk assessment tools and indicate that results derived from asymptomatic screening populations should not be directly extrapolated to hospitalized patients undergoing diagnostic evaluation.

## 1. Introduction

Lung cancer remains the leading cause of cancer-related mortality worldwide, accounting for approximately 1.8 million deaths annually [1]. Despite significant advances in diagnostic techniques and therapeutic strategies, overall survival rates remain poor, primarily because most patients are diagnosed at advanced stages [1,2]. Early detection through effective screening programs is therefore a cornerstone of lung cancer control and has the potential to substantially reduce disease-specific mortality [3].

Low-dose computed tomography (LDCT) screening has been shown to reduce lung cancer-related mortality by enabling the detection of tumors at earlier and potentially curable stages [3]. Large randomized controlled trials, including the National Lung Screening Trial (NLST) and the NELSON (Dutch-Belgian Randomized Lung Cancer Screening Trial) study, have provided robust evidence supporting the effectiveness of LDCT screening in high-risk populations [3,4]. However, widespread implementation of LDCT screening is associated with important challenges, such as increased healthcare costs, radiation exposure, false-positive findings, and overdiagnosis [5]. Consequently, accurate identification of individuals who are most likely to benefit from screening is essential to maximize clinical benefit while minimizing potential harms [6].

Current screening recommendations frequently rely on categorical eligibility criteria based on age and smoking exposure [5]. The National Comprehensive Cancer Network (NCCN) guidelines, for example, recommend LDCT screening for individuals aged 50 years or older with a smoking history of at least 20 pack-years [7]. While these criteria are straightforward and easily applicable in routine clinical practice, they do not account for substantial interindividual variability in lung cancer risk [8]. As a result, some high-risk individuals may be excluded from screening, whereas others with relatively lower risk may undergo unnecessary imaging [8].

To address these limitations, several multivariable lung cancer risk prediction models have been developed to provide individualized risk estimates [8]. Among these, the Prostate, Lung, Colorectal, and Ovarian Cancer 2012 (PLCOm2012) model and the Bach model are the most extensively validated and widely used tools [8,9]. The PLCOm2012 model estimates the 6-year risk of lung cancer by integrating demographic characteristics, smoking history, comorbid conditions, and selected personal factors [8,10]. Similarly, the Bach model provides individualized lung cancer risk estimates based predominantly on age, sex, and cumulative smoking exposure, and has been broadly applied to stratify smokers according to long-term lung cancer risk [9].

Previous studies have suggested that individualized risk models may improve the efficiency of lung cancer screening compared with simple categorical eligibility criteria [8,11]. Several comparative studies have further evaluated the performance of different lung cancer risk prediction models in screening and population-based settings. Ten Haaf et al. retrospectively validated several risk prediction models using data from the NLST and PLCO cohorts [12], while Husing and Kaaks compared risk prediction models with simplified screening eligibility criteria using German population data [13]. Katki et al. compared nine risk prediction models for selecting ever-smokers for computed tomography lung cancer screening and demonstrated that the population identified as screening eligible may vary according to the risk model applied [14]. These studies primarily focused on identifying individuals eligible for LDCT screening in asymptomatic or population-based cohorts. The clinical relevance of this analysis lies in defining the limits of model applicability. In patients already undergoing diagnostic evaluation for possible pulmonary malignancy, screening eligibility is no longer the operative clinical decision; therefore, the present study examines whether screening-oriented risk estimates retain meaningful discriminatory value when transported into this fundamentally different clinical context.

Therefore, the primary objective of this study was to compare the classification performance of two individualized lung cancer risk prediction models, PLCOm2012 and the Bach model, with NCCN guideline-based screening criteria in hospitalized patients undergoing diagnostic evaluation. The three approaches were evaluated within a common analytic cohort in which all could be assessed in the same individuals. The present study was not designed as an external validation of these approaches for their intended screening purpose or as a longitudinal evaluation of screening effectiveness. Rather, it examined how screening-oriented risk assessment strategies behave when retrospectively applied to a diagnostically enriched hospital population outside their intended-use setting, with particular emphasis on their ability to discriminate between patients with and without prevalent lung cancer. The principal contribution of the present study is therefore not a re-evaluation of established screening effectiveness, but an examination of model transportability: specifically, whether screening-oriented risk assessment strategies retain meaningful discriminatory value when applied to a diagnostically enriched hospital population that differs fundamentally from the populations in which these tools were developed and validated.

## 2. Materials and Methods

### 2.1. Study Design and Setting

This retrospective, single-center observational cohort study was conducted at the Department of Pulmonology, Yedikule Chest Diseases and Thoracic Surgery Training and Research Hospital, a tertiary referral center specializing in thoracic diseases. Consecutive adult patients hospitalized between 1 January 2024 and 1 August 2024 and undergoing diagnostic evaluation were retrospectively screened for eligibility. Lung cancer risk estimates and screening eligibility according to PLCOm2012, the Bach model, and NCCN criteria were determined retrospectively for the purposes of the present study.

This study, including the structured bedside interviews used to supplement and verify historical predictor information, was approved by the institutional ethics committee as a retrospective observational study. During the 2024 hospitalization period, eligible patients were additionally interviewed at the bedside specifically for the study using a predefined set of questions. These interviews were used to supplement and verify historical predictor information required for retrospective reconstruction of the PLCOm2012 and Bach model variables and were not part of routine clinical care or prospective outcome ascertainment. At the time of the interviews, the investigators collecting the information were unaware of the patients’ final diagnostic classification. The requirement for written informed consent, including for the structured bedside interviews, was waived by the ethics committee because the study was approved as retrospective.

### 2.2. Study Population

Consecutive adult patients hospitalized during the predefined study period were retrospectively screened for inclusion. Of 494 patients initially assessed, 254 fulfilled the study eligibility criteria and constituted the overall study cohort. Patients were excluded if clinical or demographic information required for risk assessment was unavailable, if a definitive diagnostic classification could not be established, or if pulmonary involvement represented metastatic disease from an extrapulmonary primary malignancy rather than primary lung cancer.

Primary lung cancer was defined by histopathological confirmation obtained from biopsy or surgical specimens during the index hospitalization or related diagnostic procedures. Participants classified in the non-cancer group had completed the diagnostic evaluation performed during the index hospitalization or related diagnostic work-up and had a final benign or inflammatory diagnosis, with no persistent clinical or radiological suspicion of primary lung malignancy at the conclusion of that assessment. Classification as non-cancer was therefore based on the final integrated clinical and radiological diagnostic assessment rather than solely on the absence of histopathological evidence of malignancy.

**Figure 1:**
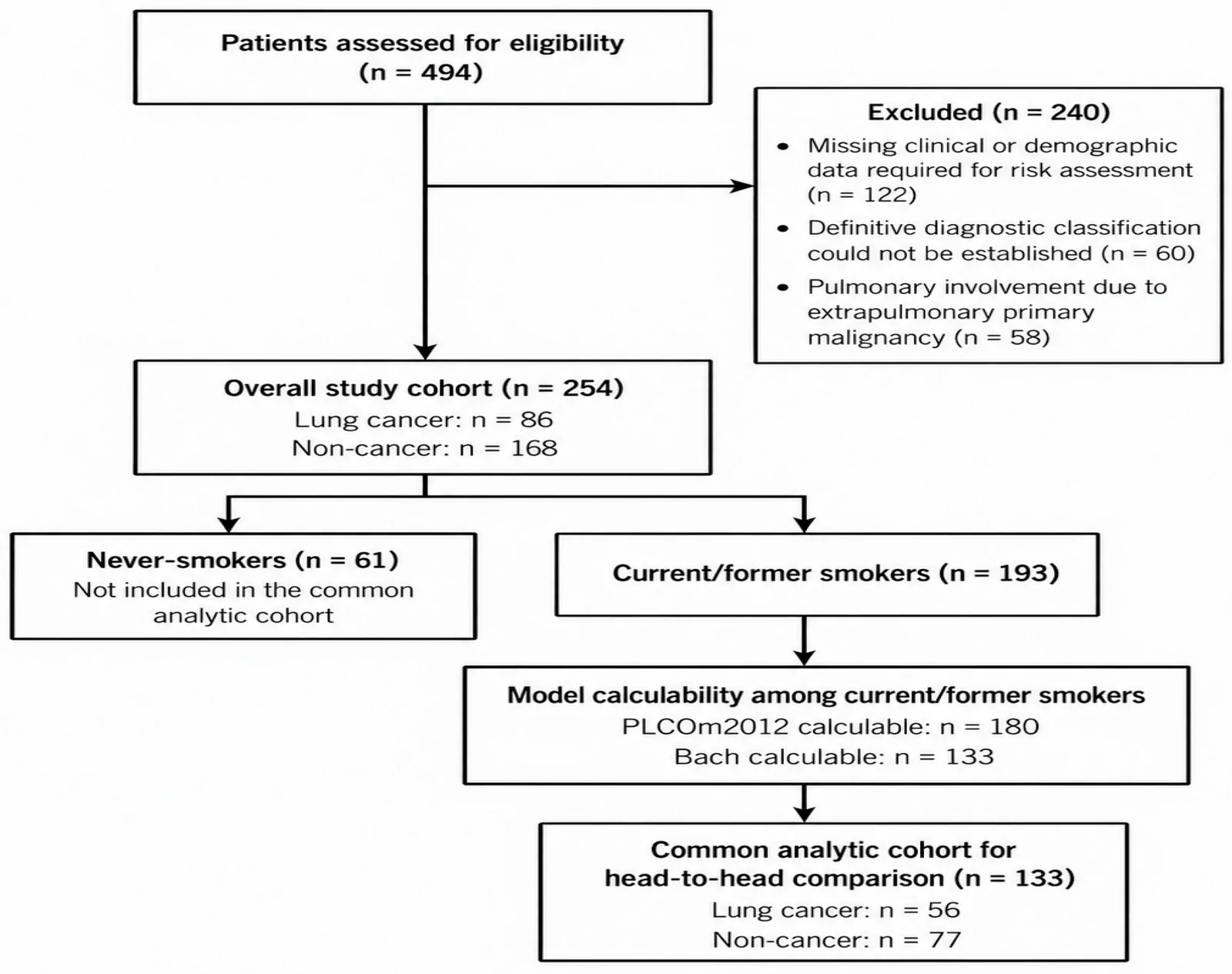
Study flowchart showing patient selection and model-specific exclusions. Model-specific non-calculability could overlap. Predictor-specific reasons for missingness were not retained separately in the analytic dataset.

Because the PLCOm2012 and Bach models require different sets of predictor variables, some patients within the overall cohort lacked individual variables required for calculation of one or both risk scores. These patients were retained in the overall study cohort but excluded from analyses involving the respective model. For the primary direct comparison of the three approaches, a common analytic cohort was defined in which PLCOm2012, Bach, and NCCN eligibility could all be determined. The study flowchart is presented in Figure 1.

### 2.3. Data Collection

Clinical, demographic, and smoking-related data were collected during the index hospitalization in 2024 using both the hospital electronic medical record system and structured bedside interviews. Historical predictor information was reconstructed for the prediction horizon of each model using available contemporaneous hospital records and structured patient interviews. For PLCOm2012, the reference baseline was six years before the index hospitalization, whereas for the Bach model it was five years before hospitalization. Only specific numerical or categorical values were used for model calculation; approximate historical estimates were not entered. Smoking-related predictors included historical smoking status, cigarettes per day, smoking duration, and cessation interval where applicable. Historical body weight was obtained from hospital records corresponding to the relevant period and confirmed with the patient during the structured interview; historical BMI was subsequently calculated using the historical body weight and recorded height. COPD history, previous cancer history, and other model-specific predictors were similarly reconstructed for the relevant baseline using available records and patient confirmation. When information was available from both sources, it was cross-checked. No imputation was performed when a required predictor was unavailable, and the corresponding model was considered non-calculable for that participant. Personal history of cancer was counted only when the diagnosis preceded the reconstructed model baseline; the current lung cancer diagnosis was not included. Smoking variables used in the analyses represented reconstructed historical values corresponding to the relevant model baseline.

Collected variables included age, sex, height, weight, body mass index (BMI), educational level, smoking status (never, former, or current smoker), smoking intensity (cigarettes per day), smoking duration (years), cumulative smoking exposure (pack-years), history of chronic obstructive pulmonary disease (COPD), personal history of cancer, family history of lung cancer, and occupational asbestos exposure.

Variables required for calculation of the PLCOm2012 and Bach risk estimates were defined according to the original published models. PLCOm2012 and Bach risk estimates were generated using publicly available web-based calculators based on the original published model algorithms, and the resulting risk estimates were recorded in the study database for analysis. Race/ethnicity for PLCOm2012 and occupational asbestos exposure for the Bach model were coded according to the categories required by the respective web-based calculators. The study flowchart, including patient selection and model-specific exclusions, is presented in Figure 1.

### 2.4. Definitions

Risk estimates for both models were calculated according to the original published equations, coefficients, and variable definitions, without local modification. The corresponding original model publications are cited to permit reproducibility. Differences in model calculability therefore reflected the specific input requirements of each model and the availability of the required predictor information.

For model calculation, the “personal history of cancer” variable referred only to a cancer diagnosis predating the current diagnostic episode; the current lung cancer diagnosis was not included in this variable.

PLCOm2012 eligibility was defined according to the estimated 6-year risk of lung cancer calculated using the original published algorithm. In accordance with previous lung cancer screening studies, a predefined risk threshold of ≥1.51% was used to classify patients as screening eligible (Table S1). The PLCOm2012 threshold of ≥1.51% was prespecified before data analysis, based on the established threshold reported in the literature, and was not derived from the present cohort. Patients with estimated risks below this threshold were classified as not eligible.

For the Bach model, the 5-year absolute risk of lung cancer was calculated using the original published algorithm and was analyzed primarily as a continuous risk estimate. Continuous discrimination was evaluated using receiver operating characteristic analysis. A cut-off value of ≥0.49% was identified from the ROC coordinates by selecting the threshold that minimized the absolute difference between sensitivity and specificity. Because this threshold was derived and evaluated in the same study population, it was not considered an externally validated screening threshold and was not used as the principal measure of Bach model performance (Table S2).

NCCN eligibility was defined according to guideline-based categorical criteria of age ≥ 50 years and a cumulative smoking exposure of ≥20 pack-years, assessed at the index hospitalization. Patients meeting the respective threshold or categorical criteria were classified as screening eligible, whereas those not meeting the criteria were classified as not eligible.

### 2.5. Outcomes

The primary outcome was the comparative classification performance of the PLCOm2012 model, the Bach model, and NCCN guideline-based screening criteria in distinguishing patients with and without prevalent primary lung cancer within the common analytic cohort. Threshold-specific performance was characterized using sensitivity, specificity, positive predictive value, negative predictive value, overall classification accuracy, and positive and negative likelihood ratios. Continuous discrimination of the PLCOm2012 and Bach risk estimates was assessed separately using ROC/AUC analysis.

### 2.6. Data Analysis and Statistical Methods

Categorical variables were presented as numbers and percentages. Continuous variables were expressed as mean ± standard deviation when normally distributed and as median with interquartile range when non-normally distributed. Comparisons between patients with and without lung cancer were performed using the chi-square test or Fisher’s exact test for categorical variables and the independent-samples t-test or Mann–Whitney U test for continuous variables, as appropriate. Comparisons presented in Table 1 were considered exploratory and were intended primarily to characterize the cohort; no adjustment for multiple comparisons was applied, and these *p* values were not used for the principal inferential conclusions of the study. For the primary head-to-head comparison of the three lung cancer risk assessment approaches, a common complete-case analytic cohort was defined comprising current or former smokers for whom PLCOm2012, Bach, and NCCN eligibility could all be determined (*n* = 133). This approach ensured that the classification performance of all three strategies was evaluated in the same individuals. Continuous discrimination of the PLCOm2012 and Bach risk estimates for prevalent lung cancer was evaluated using receiver operating characteristic (ROC) curves and area under the curve (AUC) estimates with 95% confidence intervals. Because both risk scores were evaluated in the same participants, the correlated AUCs were compared using DeLong’s method. Threshold-based analyses were considered complementary, and the cohort-derived Bach threshold was treated as exploratory. Formal calibration was not assessed because the present study evaluated prevalent lung cancer at the index hospitalization rather than prospectively observed 5- or 6-year lung cancer incidence corresponding to the prediction horizons of the original models.

Two-by-two contingency tables were constructed for each approach. Sensitivity, specificity, positive predictive value (PPV), negative predictive value (NPV), overall classification accuracy, positive likelihood ratio (LR+), and negative likelihood ratio (LR−) were calculated together with their 95% confidence intervals (CIs). Because all three threshold-based approaches were evaluated in the same participants, pairwise comparisons of sensitivity within the lung cancer subgroup and specificity within the non-cancer subgroup were performed using exact McNemar tests. Holm adjustment was applied to account for multiple pairwise comparisons. A two-sided *p* value < 0.05 was considered statistically significant. Statistical analyses were performed using IBM SPSS Statistics for Windows, version 26.0 (IBM Corp., Armonk, NY, USA).

## 3. Results

A total of 254 patients were included in the overall study cohort. At the index hospitalization in 2024, 86 patients (33.8%) had histopathologically confirmed primary lung cancer, whereas 168 patients (66.2%) had a final benign or inflammatory diagnostic outcome with no persistent clinical or radiological suspicion of primary lung malignancy. Historical predictor information corresponding to six years before the index hospitalization for PLCOm2012 and five years before the index hospitalization for the Bach model was retrospectively reconstructed as described in the Methods. Male sex (*p* < 0.001), current smoking (*p* = 0.019), a personal history of cancer (*p* = 0.001), and a family history of lung cancer (*p* < 0.001) were significantly more frequent among patients with lung cancer. Patients with lung cancer were also taller (*p* = 0.001) and had a lower BMI (*p* = 0.04). Educational background differed significantly between the groups (*p* = 0.039). Age, body weight, the presence of COPD, and asbestos exposure did not differ significantly between patients with and without lung cancer. The baseline demographic and clinical characteristics of patients with and without lung cancer are presented in Table 1.

Among the 254 participants in the overall study cohort, PLCOm2012 could be calculated in 182 (71.7%) and the Bach score in 135 (53.1%). Because both prediction models were developed primarily for smokers, we additionally examined calculability among the 193 current or former smokers. Within this group, PLCOm2012 was calculable in 180 participants (93.3%), whereas the Bach score was calculable in 133 (68.9%). These 133 participants constituted the common analytic cohort in which PLCOm2012, the Bach model, and NCCN eligibility criteria could all be evaluated in the same individuals. Of these 133 participants, 56 (42.1%) had primary lung cancer and 77 (57.9%) had a final non-cancer diagnosis.

Within the common analytic cohort, the median PLCOm2012 risk estimate did not differ significantly between patients with and without lung cancer [4.25 (IQR, 2.70–6.70) vs. 4.30 (IQR, 2.40–7.50), *p* = 0.811]. Similarly, Bach risk estimates were comparable between the two groups [0.38 (IQR, 0.19–0.73) vs. 0.45 (IQR, 0.21–0.80), *p* = 0.503]. The proportions classified as screening eligible according to PLCOm2012, the exploratory Bach threshold, and NCCN criteria also did not differ significantly between patients with and without lung cancer (Table 2).

When evaluated as continuous risk estimates, neither PLCOm2012 nor the Bach model demonstrated meaningful discrimination between patients with and without prevalent lung cancer in the common analytic cohort. The AUC was 0.488 (95% CI, 0.387–0.588) for PLCOm2012 and 0.466 (95% CI, 0.366–0.565) for the Bach model. The difference between the two correlated AUCs was not statistically significant (DeLong test, *p* = 0.545). These findings were consistent with the substantial overlap in the distributions of the continuous risk estimates between cancer and non-cancer participants (Table 3, Figure S1).

The performance characteristics of the three approaches in the common analytic cohort are shown in Table 4. PLCOm2012 demonstrated a sensitivity of 87.5% (95% CI, 76.4–93.8%) and a specificity of 15.6% (95% CI, 9.1–25.3%). The Bach approach showed lower sensitivity [42.9% (95% CI, 30.8–55.9%)] but higher specificity [57.1% (95% CI, 46.0–67.6%)]. NCCN criteria yielded the highest sensitivity [96.4% (95% CI, 87.9–99.0%)] but very low specificity [3.9% (95% CI, 1.3–10.8%)]. Positive and negative likelihood ratios were close to 1 for all three approaches, indicating limited ability to discriminate between patients with and without lung cancer in this hospital-based diagnostic cohort (Table 4). The corresponding two-by-two classification counts for each approach are provided in Table S4.

In paired threshold-specific comparisons, sensitivity differed significantly between PLCOm2012 and Bach (Holm-adjusted *p* < 0.001) and between Bach and NCCN (Holm-adjusted *p* < 0.001), whereas the difference between PLCOm2012 and NCCN was not statistically significant (Holm-adjusted *p* = 0.125). Specificity differed significantly between PLCOm2012 and Bach (Holm-adjusted *p* < 0.001), between PLCOm2012 and NCCN (Holm-adjusted *p* = 0.022), and between Bach and NCCN (Holm-adjusted *p* < 0.001).

## 4. Discussion

In this retrospective, hospital-based cohort, we examined how two established lung cancer risk prediction models, PLCOm2012 and the Bach model, together with NCCN screening eligibility criteria, behaved when applied outside their intended screening context. Importantly, the present analysis should not be interpreted as an external validation of these tools for prospective lung cancer risk prediction or screening selection. Rather, it evaluates their ability to discriminate between patients with and without prevalent lung cancer within a diagnostically enriched hospital population.

Within this context, none of the three approaches provided meaningful separation between cancer and non-cancer participants. Although PLCOm2012 and NCCN criteria classified a high proportion of cancer patients as eligible, this was accompanied by very limited specificity, while the Bach model showed a more balanced threshold-specific sensitivity–specificity profile but similarly limited discrimination. Although paired comparisons demonstrated significant differences in sensitivity and specificity between several of the threshold-based approaches, these differences did not translate into strong overall separation between patients with and without prevalent lung cancer. These findings therefore primarily illustrate the context-dependent behavior and limited transportability of screening-oriented risk assessment strategies when applied outside the populations and clinical purposes for which they were developed.

The predictive values observed in the present cohort should be interpreted with particular caution because PPV and NPV are strongly influenced by disease prevalence. The 42.1% prevalence of lung cancer in the common analytic cohort is substantially higher than that expected in an asymptomatic screening population; therefore, the PPV and NPV reported here are specific to this diagnostically enriched setting and should not be extrapolated to population-based screening. Likelihood ratios, in contrast, are not mathematically dependent on disease prevalence in the same manner. The likelihood ratios close to unity observed in the present cohort are more appropriately interpreted in relation to spectrum and selection effects and to the substantially different distribution of risk predictors in this hospital-based diagnostic population. Accordingly, these values indicate limited separation between participants with and without prevalent lung cancer within this specific clinical context rather than an effect of high disease prevalence itself.

Previous population-based screening studies have consistently shown that individualized risk prediction models may outperform simple age- and smoking-based criteria in identifying individuals who could benefit from LDCT screening [12]. Analyses derived from large screening cohorts, including PLCO- and NLST-based populations, have reported improved risk stratification and, in some settings, enhanced cancer detection using models such as PLCOm2012 or the Bach model [8,11]. Similar findings have been observed in European cohorts, including EPIC (European Prospective Investigation into Cancer and Nutrition)-Germany and other population-based studies, where the discriminatory performance of risk models was modest but generally superior to simplified eligibility criteria [13]. However, these studies were conducted in asymptomatic screening populations with substantially lower baseline lung cancer prevalence and therefore differ fundamentally from the diagnostically enriched hospital-based population evaluated in the present study.

In contrast, our cohort consisted of hospitalized patients undergoing diagnostic evaluation for a wide range of clinical indications, resulting in a substantially higher pre-test probability of lung cancer. In such settings, the operating characteristics of screening-oriented risk assessment tools may differ considerably from those observed in asymptomatic screening populations. The markedly reduced specificity observed for PLCOm2012 and NCCN criteria in our common analytic cohort likely reflects the challenge of applying tools developed for population-level screening to a diagnostically enriched population with heterogeneous clinical characteristics and elevated baseline risk. Our findings are therefore consistent with prior observations that the relative performance of risk-based approaches may vary according to disease prevalence, population characteristics, and clinical decision thresholds [13,15].

An important distinction is that the present analysis does not reproduce the longitudinal structure of an organized lung cancer screening program. Prospective screening studies enroll defined at-risk populations and follow them through serial screening rounds, thereby capturing cancers arising during the observation period [3,4]. In contrast, our cohort was assembled among patients hospitalized in 2024. Individuals who may have been at risk at the reconstructed model baseline but developed lung cancer, underwent treatment, died, or otherwise did not enter the present hospital cohort during the intervening five- to six-year period were necessarily not represented. Therefore, the present study cannot estimate screening yield, interval cancer incidence, mortality benefit, or the effectiveness of contemporary screening protocols. Rather, reconstruction of historical predictor values was undertaken solely to permit application of the original risk algorithms and to examine their transportability to a diagnostically enriched clinical population.

High sensitivity alone should not be considered sufficient evidence of an effective screening strategy. Screening requires a balance between identifying individuals who may benefit from early detection and limiting unnecessary downstream evaluation. False-positive screening findings may lead to additional imaging, invasive procedures, psychological burden, radiation exposure, and increased healthcare utilization [5]. In the present cohort, the very high sensitivity of NCCN criteria was accompanied by classification of nearly the entire common analytic population as eligible and therefore extremely low specificity. Nearly all participants in the common analytic cohort (96.2%) met NCCN screening eligibility criteria, which is unsurprising in a population restricted to current or former smokers already undergoing thoracic diagnostic evaluation. Therefore, the observed specificity of 3.9% should not be interpreted as evidence of intrinsically poor screening performance of NCCN criteria. Rather, it indicates that these broad eligibility criteria provided little separation between patients with and without prevalent lung cancer within this highly selected hospital population. Similarly, PLCOm2012 achieved high sensitivity but provided limited separation between cancer and non-cancer participants. Importantly, the specificity estimates reported here relate to risk-based eligibility classification in this selected hospital cohort and should not be equated with the false-positive rate of LDCT itself. The Bach model showed lower sensitivity but higher specificity, producing a more balanced sensitivity–specificity profile; however, its likelihood ratios remained close to unity, indicating limited overall discrimination. This may partly reflect its reliance on a more restricted set of predictors, primarily age, sex, and smoking exposure, which may be insufficient to capture the heterogeneity of risk in a hospital-based diagnostic population [9].

Recent real-world studies further support the context-dependent nature of lung cancer risk assessment [13,16]. Community-based screening evaluations, including those conducted within UK Lung Health Check programs, have shown that the performance of risk models varies substantially according to population characteristics and selected risk thresholds [17]. Importantly, these studies also emphasize practical challenges associated with implementing complex risk algorithms in routine clinical settings, including data availability and model applicability [16,17]. Real-world implementation studies have further demonstrated variability in screening eligibility, guideline concordance, and screening outcomes across different clinical practice settings [18,19,20].

From a clinical perspective, the present findings should not be interpreted as evidence that PLCOm2012, the Bach model, or NCCN criteria perform inadequately within organized lung cancer screening programs [21,22]. Rather, they demonstrate that screening eligibility and future-risk prediction do not necessarily retain the same discriminatory meaning when transported into a population already undergoing diagnostic evaluation. Screening-oriented risk tools should therefore not be used as surrogates for diagnostic assessment in patients with suspected pulmonary malignancy, and their outputs should always be interpreted within the clinical context for which the tools were developed [23].

Several limitations of this study should be acknowledged. First, the retrospective, single-center design and the inclusion of hospitalized patients undergoing diagnostic evaluation may limit the generalizability of the findings and introduce selection and spectrum bias. The prevalence of lung cancer in this cohort was substantially higher than that typically observed in asymptomatic screening populations, limiting direct comparison with population-based screening studies. Second, retrospective reconstruction of predictor information five to six years before hospitalization remains an important source of measurement uncertainty. Although only specific numerical or categorical values were used and available historical records were cross-checked with patient information, these predictors were not prospectively collected at the original model baseline and could not be independently validated against a prospective reference standard. In addition, predictor-level provenance was not retained in the analytic dataset; therefore, the relative contribution of contemporaneous documentation and patient confirmation could not be quantified, and a sensitivity analysis restricted exclusively to objectively documented historical predictors was not feasible. Third, model-specific predictor information was incomplete in a substantial proportion of patients, particularly for the Bach model. To improve comparability, the primary head-to-head analysis was therefore restricted to a common complete-case–cohort in which all three approaches could be evaluated. Restriction of the primary comparison to participants with calculable results for all three approaches ensured identical denominators but may have introduced complete-case selection bias. Participants included in the common analytic cohort differed from excluded ever-smokers in several characteristics, particularly smoking duration and intensity, smoking status, sex distribution, and asbestos exposure (Table S3). Although lung cancer prevalence did not differ significantly between the two groups, these differences indicate that the common analytic cohort should not be assumed to be fully representative of the broader hospitalized smoking population. The relatively small common analytic cohort also limited the precision of the estimated performance measures. Although the overall pattern of findings was consistent, several estimates were accompanied by relatively wide 95% confidence intervals. Larger multicenter cohorts would provide narrower confidence intervals and more stable estimates and would permit adequately powered subgroup analyses. Fourth, the Bach threshold of 0.49% was derived and evaluated within the same study population and should therefore be considered exploratory; its performance may be optimistic in the absence of internal or external validation. Detailed cancer stage and uniformly classified histological subtype were not available for all participants and therefore could not be analyzed reliably. Finally, although participants classified as non-cancer had a final benign or inflammatory diagnostic assessment with no persistent clinical or radiological suspicion of primary lung malignancy, standardized prospective follow-up was not available for all participants. Therefore, delayed or subsequently diagnosed lung cancer cannot be completely excluded, introducing the possibility of outcome misclassification in the non-cancer reference group.

## 5. Conclusions

In this diagnostically enriched hospital cohort, PLCOm2012, the Bach model, and NCCN screening eligibility criteria showed limited ability to discriminate between patients with and without prevalent lung cancer, although this finding should be interpreted in light of patient selection and uncertainty related to retrospective reconstruction of historical predictor data. These findings should not be interpreted as evidence against the validity or effectiveness of these approaches in their intended screening populations. Rather, they illustrate the context-dependent loss of discriminatory utility that may occur when screening-oriented risk assessment strategies are transported into a clinical population with a fundamentally different case-mix and diagnostic purpose. Screening eligibility and future-risk estimates should therefore not be interpreted as diagnostic surrogates in patients already undergoing evaluation for possible lung malignancy.

## Figures and Tables

**Table 1:** Baseline demographic and clinical characteristics of patients with and without lung cancer.

Variables	All Patients (*n* = 254)	Cancer (*n* = 86)	Non-Cancer (*n* = 168)	*p*
Age, years	63.6 ± 11	62.4 ± 9.5	64.2 ± 11.6	0.21
Female sex, *n* (%)	79 (31.1)	14 (16.3)	65 (38.7)	<0.001
Height, cm	168.2 ± 8.6	170.7 ± 7.5	166.9 ± 8.8	0.001
Weight, kg	74.3 ± 16.6	73.8 ± 13.6	74.5 ± 17.9	0.75
BMI, kg/m^2^	26.2 ± 5.4	25.2 ± 4	26.7 ± 5.9	0.04
Smoking, *n* (%)				0.019
Never smoked	61 (24)	12 (14)	49 (29.2)	
Former smoker	80 (31.5)	28 (32.6)	52 (31)	
Current smoker	113 (44.5)	46 (53.5)	67 (39.9)	
Cigarettes per day	27.5 ± 14.1	28.6 ± 15.2	26.8 ± 13.3	0.37
Smoking, years	35.1 ± 12	36.1 ± 10.5	34.5 ± 12.9	0.37
COPD, *n* (%)	104 (40.9)	36 (41.9)	68 (40.5)	0.83
Educational background				0.039
Illiterate	44 (17.3)	9 (10.5)	35 (20.8)	
Primary school graduate	161 (63.4)	52 (60.5)	109 (64.8)	
High school graduate	40 (15.7)	19 (22.1)	21 (12.5)	
University graduate	7 (2.8)	5 (5.8)	2 (1.2)	
Doctorate	2 (0.8)	1 (1.2)	1 (0.6)	
Personal history of cancer, *n* (%)	28 (11)	17 (19.8)	11 (6.5)	0.001
Family history of lung cancer, *n* (%)	100 (39.4)	49 (57)	51 (30.4)	<0.001
Asbestos exposure, *n* (%)	61 (24)	23 (26.7)	38 (22.6)	0.46

BMI: body mass index; COPD: chronic obstructive pulmonary disease; SD: standard deviation. Parameters that follow a normal distribution are presented as mean ± standard deviation. *p* values are unadjusted and are presented for descriptive, exploratory characterization of the cohort. Smoking intensity and smoking duration represent retrospectively reconstructed historical smoking exposure corresponding to the model-specific reference period rather than values recorded at the index hospitalization.

**Table 2:** Lung cancer risk estimates and screening eligibility according to PLCOm2012, Bach, and NCCN criteria in the common analytic cohort.

Variables	All Patients (*n* = 133)	Lung Cancer (*n* = 56)	Non-Cancer (*n* = 77)	*p*
PLCOm2012 risk	4.30 (2.60–7.30)	4.25 (2.70–6.70)	4.30 (2.40–7.50)	0.811
PLCOm2012 screening eligible, *n* (%)	114 (85.7)	49 (87.5)	65 (84.4)	0.803
Bach 5-year risk	0.45 (0.20–0.75)	0.38 (0.19–0.73)	0.45 (0.21–0.80)	0.503
Bach screening eligible, *n* (%)	57 (42.9)	24 (42.9)	33 (42.9)	1.000
NCCN screening eligible, *n* (%)	128 (96.2)	54 (96.4)	74 (96.1)	1.000

PLCOm2012: Prostate, Lung, Colorectal, and Ovarian Cancer 2012; NCCN: National Comprehensive Cancer Network. Data are presented as median (interquartile range) or *n* (%), as appropriate. PLCOm2012 screening eligibility was defined using the predefined ≥1.51% 6-year risk threshold. Bach screening eligibility was defined using the exploratory cohort-derived 5-year risk threshold of ≥0.49%.

**Table 3:** Continuous discrimination of PLCOm2012 and Bach risk estimates for prevalent lung cancer in the common analytic cohort.

Model	AUC	95% CI
PLCOm2012	0.488	0.387–0.588
Bach	0.466	0.366–0.565

AUC: area under the receiver operating characteristic curve; CI: confidence interval. The correlated AUCs were compared using DeLong’s method (*p* = 0.545).

**Table 4:** Performance characteristics of PLCOm2012, Bach, and NCCN criteria in the common analytic cohort.

	Sensitivity, % (95% CI)	Specificity, % (95% CI)	PPV, % (95% CI)	NPV, % (95% CI)	Overall Classification Accuracy, %	LR+ (95% CI)	LR− (95% CI)
PLCOm2012	87.5 (76.4–93.8)	15.6 (9.1–25.3)	43.0 (34.3–52.2)	63.2 (41.0–80.9)	45.9 (37.6–54.3)	1.04 (0.90–1.19)	0.80 (0.34–1.91)
Bach	42.9 (30.8–55.9)	57.1 (46.0–67.6)	42.1 (30.2–55.0)	57.9 (46.7–68.4)	51.1 (42.7–59.5)	1.00 (0.67–1.49)	1.00 (0.74–1.35)
NCCN criteria	96.4 (87.9–99.0)	3.9 (1.3–10.8)	42.2 (34.0–50.8)	60.0 (23.1–88.2)	42.9 (34.8–51.4)	1.00 (0.94–1.07)	0.92 (0.16–5.31)

PLCOm2012: Prostate, Lung, Colorectal, and Ovarian Cancer 2012; NCCN: National Comprehensive Cancer Network. PPV: positive predictive value; NPV: negative predictive value; LR+: positive likelihood ratio; LR−: negative likelihood ratio; CI: confidence interval. Bach estimates were based on the exploratory cohort-derived, 5-year risk threshold of ≥0.49%. PPV and NPV are prevalence-dependent and should be interpreted in the context of the high lung cancer prevalence in this diagnostically enriched cohort. Pairwise comparisons of sensitivity and specificity were performed using exact McNemar tests with Holm adjustment for multiple comparisons. Holm-adjusted *p* values for sensitivity were <0.001 for PLCOm2012 vs. Bach, 0.125 for PLCOm2012 vs. NCCN, and <0.001 for Bach vs. NCCN. Corresponding adjusted *p* values for specificity were <0.001, 0.022, and <0.001, respectively.

## Data Availability

De-identified participant-level data underlying the findings of this study are not publicly available but may be obtained from the corresponding author upon reasonable request.

## Supplementary Materials

The following supporting information can be downloaded at https://www.mdpi.com/article/10.3390/healthcare14193350/s1, Figure S1: Receiver operating characteristic curves of the continuous PLCOm2012 and Bach risk estimates for discrimination of prevalent lung cancer in the common analytic cohort (*n* = 133); Table S1: Variables included in the PLCOm2012 lung cancer risk prediction model; Table S2: Variables included in the Bach lung cancer risk prediction model; Table S3: Comparison of current/former smokers included in and excluded from the common analytic cohort; Table S4. Two-by-two classification counts in the common analytic cohort.

## Abbreviations

LDCT	low-dose computed tomography
NLST	National Lung Screening Trial
NCCN	National Comprehensive Cancer Network
PLCOm2012	Prostate, Lung, Colorectal, and Ovarian Cancer 2012
BMI	body mass index
COPD	chronic obstructive pulmonary disease
ROC	receiver operating characteristic
PPV	positive predictive value
NPV	negative predictive value
