# Supplementary material for: Beyond Screening Populations: Context-Dependent Performance of Lung Cancer Risk Assessment Strategies in a Diagnostically Enriched Cohort
Source: Healthcare (Basel). 2026 Oct 8;14(19):3350. doi: 10.3390/healthcare14193350 (PMC13665566; doi:10.3390/healthcare14193350)
Supplement: Supplementary file 1 [file healthcare-14-03350-s001.zip › healthcare-4572361-supplementary.pdf]

Supplementary Table S1. Variables included in the PLCOm2012 lung cancer risk prediction model.

Variable	Description
Age	Age at the time of risk assessment (years)
Sex	Biological sex (male/female)
Smoking status	Current or former smoker
Smoking intensity	Average number of cigarettes smoked per day
Smoking duration	Total duration of smoking (years)
Time since smoking cessation	Years since quitting smoking (former smokers only)
Body mass index	Calculated as weight (kg) divided by height squared (m^2)
History of chronic obstructive pulmonary disease (COPD)	Physician-diagnosed COPD (yes/no)
Personal history of cancer	History of any cancer diagnosis excluding non-melanoma skin cancer (yes/no)
Family history of lung cancer	First-degree relative with lung cancer (yes/no)
Educational level	Highest attained level of education
Race/ethnicity	Coded according to the categories available in the web-based PLCOm2012 calculator

Notes: Individual 6-year lung cancer risk estimates were generated using a publicly available web-based calculator based on the original PLCOm2012 risk prediction model. Eligibility for LDCT screening was defined using a risk threshold of ≥1.51%.

Supplementary Table S2. Variables included in the Bach lung cancer risk prediction model.

Variable	Description
Age	Age at the time of risk assessment (years)
Sex	Biological sex (male/female)
Smoking status	Current or former smoker
Smoking intensity	Average number of cigarettes smoked per day
Smoking duration	Total duration of smoking (years)
Cumulative smoking exposure	Calculated smoking exposure based on duration and intensity
Time since smoking cessation	Years since quitting smoking (former smokers only)
Occupational asbestos exposure	Coded as present or absent according to occupational history

Notes: Individual lung cancer risk estimates were generated using a publicly available web-based calculator based on the original Bach risk prediction model. In the present study, the cut-off value applied for LDCT screening eligibility was derived from receiver operating characteristic (ROC) curve analysis performed in the study cohort and was treated as exploratory.

Figure S1. Receiver operating characteristic curves of the continuous PLCOm2012 and Bach risk estimates for discrimination of prevalent lung cancer in the common analytic cohort (n = 133).

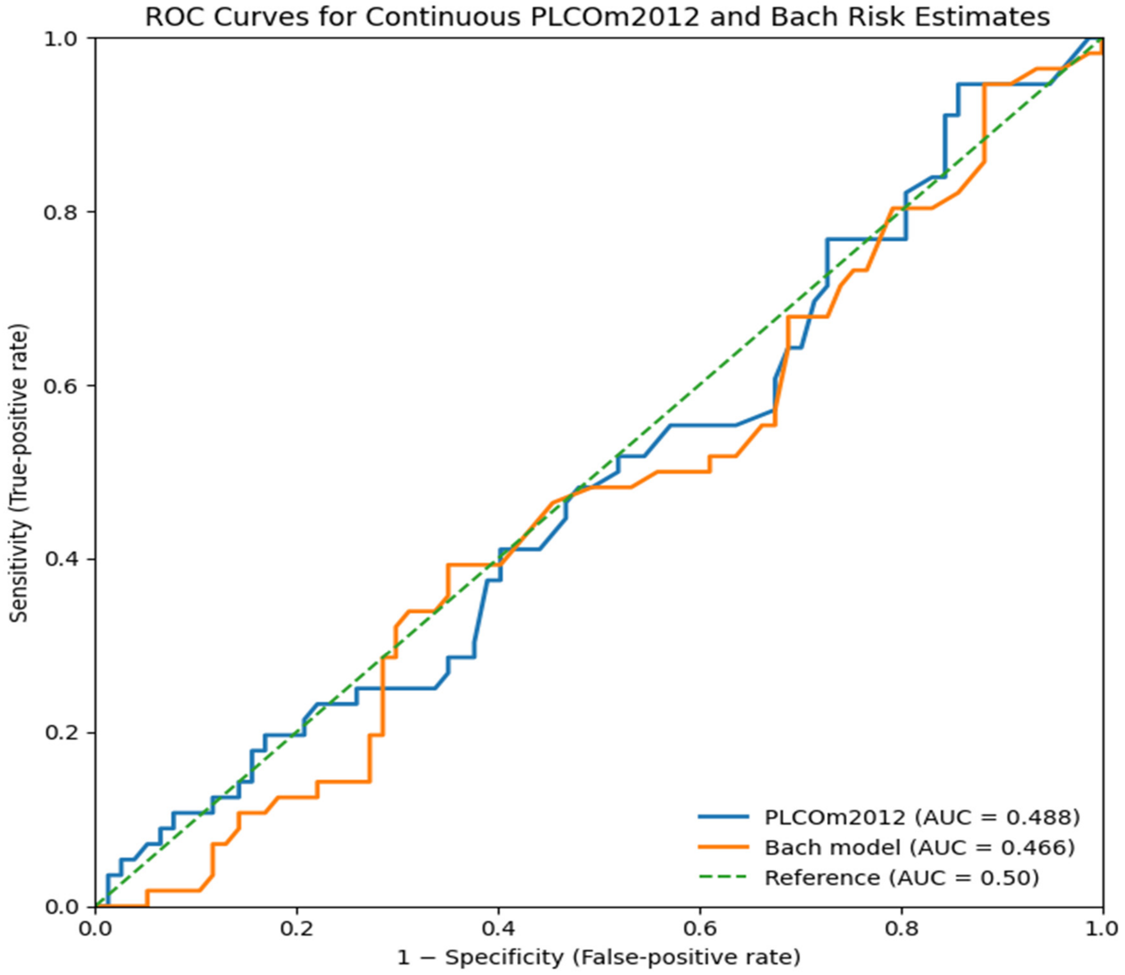

Supplementary Table S3. Comparison of current/former smokers included in and excluded from the common analytic cohort.

Variable	Included ever smokers (n = 133)	Excluded ever-smokers (n = 60)	p
Age, years	62.5 ± 6.9	62.1 ± 15.1	0.836
Female sex, n (%)	15 (11.3)	15 (25.0)	0.026
Current smoker, n (%)	86 (64.7)	27 (45.0)	0.016
Smoking duration, years	38.5 ± 9.2	27.8 ± 14.4	<0.001
Cigarettes per day	29.8 ± 13.6	22.6 ± 14.2	0.001
COPD, n (%)	75 (56.4)	26 (43.3)	0.127
Previous cancer, n (%)	14 (10.5)	6 (10.0)	1.000
Family history of lung cancer, n (%)	57 (42.9)	22 (36.7)	0.515
Asbestos exposure, n (%)	22 (16.5)	19 (31.7)	0.029
Lung cancer, n (%)	56 (42.1)	18 (30.0)	0.150

Data are presented as mean ± standard deviation or n (%), as appropriate. Included participants were current or former smokers with calculable PLCOm2012, Bach, and NCCN results. Excluded participants were current or former smokers who could not be included in the common analytic cohort because at least one of the three approaches was non-calculable. COPD: chronic obstructive pulmonary disease.

Supplementary Table S4. Two-by-two classification counts in the common analytic cohort.

Approach	TP	FN	TN	FP
PLCOm2012	49	7	12	65
Bach model	24	32	44	33
NCCN criteria	54	2	3	74

TP: true positive; FN: false negative; TN: true negative; FP: false positive. Counts are based on the common analytic cohort (n = 133), including 56 participants with prevalent lung cancer and 77 participants without lung cancer. Bach classification was based on the cohort-derived exploratory threshold of ≥0.49%.
